# On the Chloride Distribution in Concrete and Mortar Samples after an RCM Test

**DOI:** 10.3390/ma16082952

**Published:** 2023-04-07

**Authors:** Hannah Drenkard, Christian Fischer, Veit Sauer, Christoph Gehlen

**Affiliations:** 1 Faculty of Architecture and Civil Engineering, Technical University of Applied Sciences Würzburg-Schweinfurt, Röntgenring 8, 97070 Würzburg, Germany; christian.fischer@thws.de; 2 Centre for Building Materials, Department of Materials Engineering, TUM School of Engineering and Design, Technical University of Munich, Franz-Langinger-Straße 10, 81245 München, Germany; 3 F.A. Finger-Institute for Building Materials Science, Faculty of Civil Engineering, Bauhaus-University Weimar, Coudraystr. 11, 99423 Weimar, Germany; veit.sauer@uni-weimar.de

**Keywords:** chloride profile, RCM test, drill powder, concrete, mortar, chloride migration coefficient, chloride ingress front

## Abstract

It is of essential need to face the challenges of CO_2_ reduction in industrial cement and concrete production reliable test methods in order to evaluate the performance of concretes, especially with regard to the service life of our infrastructure. The rapid chloride migration test (RCM test) is a standard method to assess the resistance against chloride ingress of concrete. However, during our study, certain critical questions arose with regard to the chloride distribution. The sharp chloride ingress front based on the model assumptions contradicted the shallow gradient of the experimental data. For this reason, investigations on chloride distribution in concrete and mortar samples after RCM tests were performed. The focus was on the factors influencing the extraction, e.g., time after RCM test and the location on sample. Furthermore, differences between concrete and mortar samples were investigated. The investigations showed that no sharp gradient on concrete samples was found due to the extremely uneven chloride front. In contrast, the theoretical profile shape was instead demonstrated on mortar specimens. The prerequisite for this result is that the drill powder must be taken directly after the completion of the RCM test from very uniform penetration areas. Thus, the model assumptions on the chloride distribution via the RCM test could be confirmed.

## 1. Introduction

Corrosion of the reinforcement, due to chloride penetration, is one of the most common causes of damage to existing concrete structures exposed to seawater or deicing salt. To evaluate the performance of the concretes utilized for infrastructure projects, concrete material must be tested against chloride penetration. The parameter determined in this context is the chloride diffusion coefficient. This is a key parameter for calculating the service life of chloride-loaded structures [1]. Besides long-term diffusion tests, the rapid chloride migration test (RCM test) is an important tool to evaluate the resistance against chloride ingress and therefore in the evaluation of the durability of concrete.

Originally, the RCM test was developed by Tang and Nilsson [2] and was called the CTH test in the early 1990s. At the beginning of the 2000s, the method was accepted as a Nordic standard NT BUILD 492 [3] and as BAW Merkblatt, 2004 [4] (with continuous further development), in Germany. The RCM test method has decisive advantages compared to other testing methods as a chloride diffusion test. It is faster: generally, 24 to 48 h compared to 90 days, and less effort is needed to perform the test. Furthermore, compared to other accelerated tests as the coulomb test (which accounts for all charged ions, not only chlorides) or the steady state migration test, the RCM test is similar, less complex, and more precise [5].

The theoretical background of Tang’s method is based on a relationship between diffusion and migration for calculating the chloride diffusion coefficient (herein, as in most of the literature, will be referred to as the chloride migration coefficient *D_RCM_*) from the non-steady state migration test—the RCM test. The non-steady state process is characterized by Tang using the following equation:

(1) $$\frac{\partial c}{\partial t}=D_{RCM}\cdot \left(\frac{\partial^{2}c}{\partial x^{2}}-\frac{zFU}{RTL}\cdot \frac{\partial c}{\partial x}\right)$$

where *c* is the free chloride concentration at depth *x*, time *t,* and temperature *T; U* is the driving voltage of the electrical field through the specimen with thickness *L*; the constants are *z* = ion charge of chlorides, *F* = Faraday constant, and *R* = gas constant; *D_RCM_* is the searched parameter, the chloride migration coefficient; and *c* denotes the free chloride concentration affected by chloride binding. For certain experimental conditions at migration tests, a constant chloride binding can be assumed [2]. 

Tang used the following analytical solution for Equation (1):

(2) $$c=\frac{c_{0}}{2}\cdot \left[e^{ax}\cdot erfc\left(\frac{x+aD_{RCM}\cdot t}{2\sqrt{D_{RCM}\cdot t}}\right)+erfc\left(\frac{x-aD_{RCM}\cdot t}{2\sqrt{D_{RCM}\cdot t}}\right)\right],$$

where *c_0_* is the concentration of chlorides in bulk solution; *a* is the *zFU/RTL;* and *erfc* is the complement of the error function *erf* (*erfc* = 1 − *erf*). During a standard RCM test, parameters *U* and *x* are large enough; in fact, term $erfc\left(\frac{x+aD_{RCM}\cdot t}{2\sqrt{D_{RCM}\cdot t}}\right)$ turns 0 and Equation (2) turns into:

(3) $$c=\frac{c_{0}}{2}\cdot erfc\left(\frac{x-aD_{RCM}\cdot t}{2\sqrt{D_{RCM}\cdot t}}\right)$$

**Figure 1:**
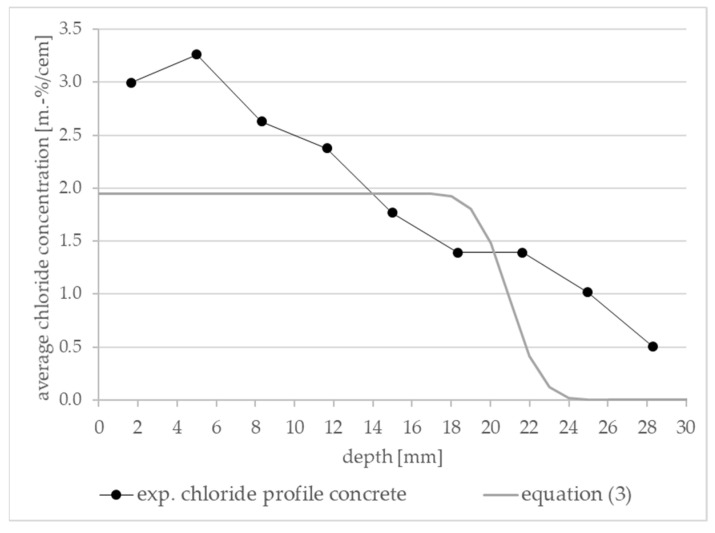
Comparison of an exemplary experimental chloride profile (own investigation of a concrete sample) and the theoretical prediction based on Tang [2], Equation (3).

Although not a standard in the testing procedure of the RCM test and other migration tests, the chloride distribution after the test was investigated [6,7,8,9,10,11,12]. Some scientists compared the actual chloride distribution to the above described model assumptions and found differences. Figure 1 shows the chloride distribution within an exemplary concrete sample and the corresponding model assumption. 

The discrepancy was already discussed by Stanish and Spiesz [7,10]. Stanish [7] investigated six different concrete mixtures with comparatively low w/c ratios (0.35–0.45) and generated 66 chloride distribution profiles after RCM tests. In all profiles, the chloride concentration decreased gradually. The profiles did not show the characteristics of the model: (i) a plateau close to the ingress surface and (ii) followed by a sharp decrease close to the line of color change of the indicator (see Figure 1, Equation (3) curve). Spiesz et al. [10] investigated a mortar mixture with high cement content c = 785.6 kg/m^3^, and a low w/c ratio of 0.26 at different applied voltages, *U*, between 35 V and 60 V. These profiles differed from the model assumptions in the same way as the profiles reported by Stanish [7]. Tang [2] also compared the theoretical chloride profiles of the model with experimental data from the ordinary Portland cement (OPC) and mortar paste specimens with w/c ratios between 0.4 and 0.8. The results also showed a less sharp gradient of the experimental data. Tang provided the following possible reasons for that: (i) different pore distributions result in different penetration behavior, and thus in different profile shapes; (ii) the rate of chloride binding influences the shape of profiles and not the penetration depth; and (iii) the presence of other ions (OH^−^, Na^+^, K^+^, Ca^2+^, etc.) and their movement in the electrical field influence the chloride binding and thus the shape of the profiles [2]. 

However, two research teams found experimental steep gradients of chloride distribution comparable to the theoretical assumptions [11,12]. Voinitchi et al. performed migration tests at mortar samples with an OPC content of c = 618 kg/m^3^ and a w/c ratio of 0.43. The resulting chloride profiles after a two-day test showed a plateau close to the ingress surface, followed by a sharp decrease in the curve, comparable to the model assumption [11]. Baroghel-Bouny et al. also performed migration tests at two different concrete mixtures with a maximum grain size of 20 mm, a cement content of c = 230/353 kg/m^3^, w/c ratios of 0.84/0.49, a voltage of *U* = 10 V and test durations of *t* = 16 h or 30 h. The resulting chloride profiles also showed the attributes of the profiles of the model [12]. The migration tests performed by Voinitchi et al. and Baroghel-Bouny et al. were also non-steady state tests. However, they differed from the standard RCM test by the use of two parallel cells (upstream and downstream) where the sample was fixed in between, as comparable to NT Build 355 setup [13].

No uniform picture can be derived from the reported results. However, against the background of CO_2_ reduction in the cement industry and newly emerging cements, the reliability of the test method is of crucial importance. For this reason, the purpose of this publication is to address the question of chloride distribution after an RCM test and its comparison to the theoretical chloride profiles based on the model assumptions. This is essential since the assumption of the determination of the non-steady state chloride diffusion coefficient in the context of a migration experiment is based on it. In addition, it should be clarified to what extent the type, timing, and exact location of the drill powder extraction have an influence on the profile course. 

## 2. Materials and Methods

### 2.1. Materials and RCM Test Setup

In this section, the materials used and, in particular, the test procedures are described in detail as the individual steps are decisive for the result. The following cements were used to prepare the concrete and mortar samples: CEM III/A 42.5 N, CEM II/A-LL 42.5 N, and CEM I 42.5 N (all compliant to DIN EN 197-1 [14]). CEM I was chosen as a reference, as the RCM test was originally developed on this type, CEM II/A-LL as a cement very frequently used in practice, and CEM III/A as a cement frequently used for structures with chloride exposure. The concrete/mortar mix designs as well as the compressive strength according to DIN EN 12390-3 [15] and DIN EN 196-1 [16], respectively, at the age of 28 days are shown in Table 1. 

The general sample preparation and RCM test setup was according to the German “BAW Merkblatt Dauerhaftigkeitsbemessung und –bewertung von Stahlbetonbauwerken bei Carbonatisierung und Chlorideinwirkung (MDCC)” [17]. For the investigation, cubes with 150 × 150 × 150 mm^3^ as well as cylinders of 100 mm diameter and 200 mm height were produced and demolded after 24 h. Subsequently, they were stored under water for another 27 days. For the cubes, a few days before the test, a cylinder with diameter 100 mm was drilled out of the center of the cube, and a 10 mm thick slice was cut from the surface. For the cylinders, only the 10 mm thick slice was cut from the surface. Afterwards, both types of specimens with a height of 50 mm were then sawn off and ground to smoothen the surfaces. After preparation, the specimens were stored under water again until the start of the test. In contrast to NT Build 492 [3], BAW Merkblatt [17] does not provide for vacuum saturation. 

**Figure 2:**
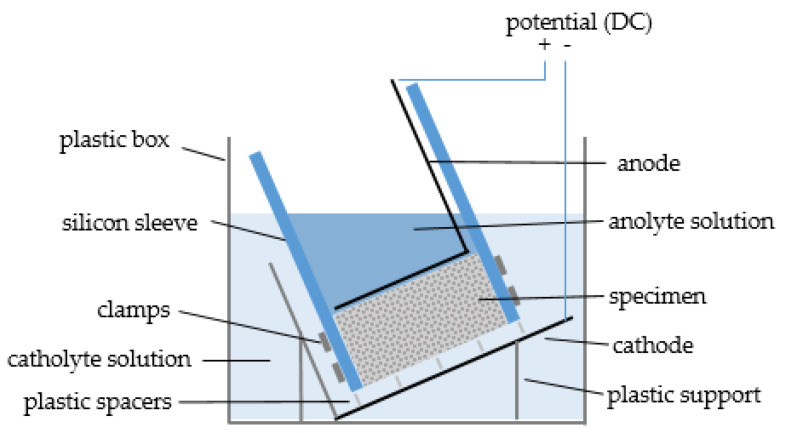
Theoretical scheme of the RCM setup.

The used catholyte reservoir contained 10% NaCl in 0.2 mol/L KOH, and the anolyte solution filled in the sleeves above the specimen was 0.2 mol/L KOH. Figure 2 shows the theoretical scheme of the RCM setup. 

Deviating from the BAW Merkblatt test duration for all CEM III/A specimens was extended (48 h instead of 24 h) to reach an appropriate penetration depth (Table 1). After the test, the specimens were split into two halves, A and B. On these freshly split halves, analyses of the free chloride penetration front and the chloride penetration depth x_d_ were carried out with 0.1 mol/L AgNO_3_ and ultraviolet light by using caliper and ruler, respectively. Potassium chromate/dichromate or fluoresceine were not used. It is sometimes applied to clarify the penetration front, especially for dark binders such as ground granulated blast furnace slag. 

### 2.2. Extraction of Drill Powder

**Figure 3:**
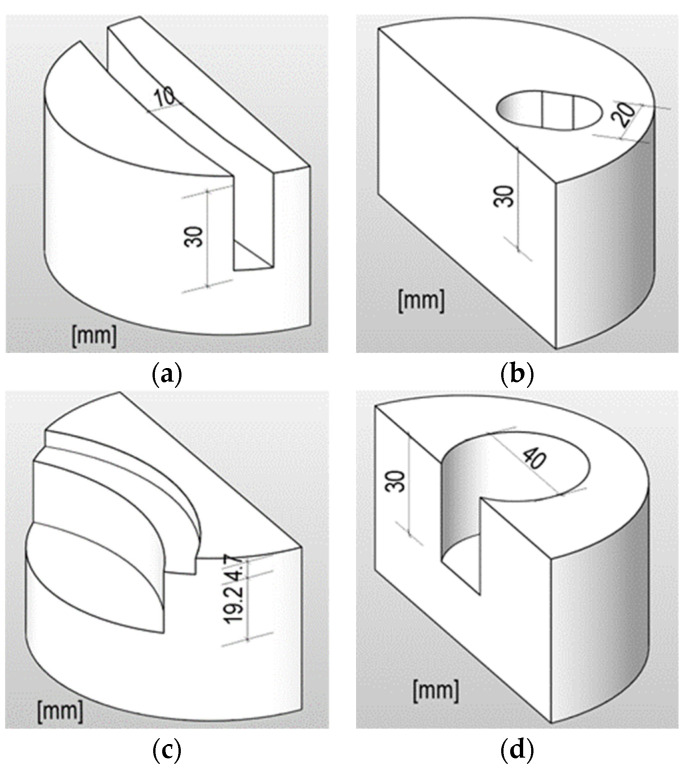
Geometries of the concrete powder samples after extraction (dimensions in mm); extraction method: (**a**) slot grinding; (**b**) oblong drill hole; (**c**) disc grinder; (**d**) wide drill hole.

The drill powder of the specimen halves was extracted using different methods (Figure 3). Different methods had pros and cons, and in fact, a development took place over time. Developments in the extraction methods were made parallel with the chloride analysis of the samples. The further developments were carried out in response to certain influences on the analysis results as well as the amount of drill powder. After performing all extraction methods, method (d) was finally found to be the best method with the lowest influence of edge effects (see Section 3.3.2) at an adequate amount of drill powder. In addition, with this method, the ablation area is large enough so that eventual pores or aggregates have less influence on the chloride concentration than with a single borehole (as with method (b), for example).

For all removal methods, the specimens were clamped in a column drill with depth stop. By presetting the depth interval, it was thus possible to grind out the drill powder with different chloride concentrations at defined layer intervals. The grinding of the surfaces removed both the binder matrixes and the aggregates from the specimen. All procedures were applied dry to avoid mixing of the individual layers. After each grinding interval, it was observed that before the next interval, the grinding site should be thoroughly cleaned by brush and vacuum cleaner, so that the next layer would not be contaminated with previous drill powder. 

Method (a): The first method used a diamond-tipped drill bit with a diameter of 10 mm to remove the drill powder by approx. 3.33 mm per interval step by turning the specimen clamping sideways. This results in a radial slot profile across the entire width of the specimen half. It turned out that the chloride concentrations were influenced by possible edge effects in the edge area (see Section 3.3.2). In addition, it was challenging to create a slot when turning sideways. This method produced adequate amounts of drill powder.

Method (b): Similar drill bit with a diameter of 20 mm was used here. To increase the removal area, the drill hole was widened by swiveling the specimen clamping sideways. The position of the drill hole was at the edge of the specimen half near the formed surface and the fracture surface after indicator impact. Due to the swiveling, two drillings per specimen were necessary. Areas with possible edge effects were not considered in this method, but the amount of drill powder produced was relatively small.

Method (c): The grinding tool used was a wide disc grinding head, which is set with synthetic diamonds. The ground surface is located on the switched side of the specimen half due to the grinding disc geometry. Due to the smaller intervals on the upper side of the specimen half, a wider area was initially removed to generate the same amount of drill powder. In the underlying layers, the grinding area was finally reduced since the larger intervals generate more drill powder per area. A sufficient amount of drill powder was produced, but the chloride concentrations were also affected by areas with possible edge effects similar to method (a).

Method (d): In this method, the drill powder of the individual layers was removed from the center of the previous cylindrical sample using a wider drill bit with a diameter of 40 mm. The fixation of the drill bit could be maintained until the last layer, so that the individual layer depths can be precisely set. Chloride concentrations were not affected by areas with possible edge effects and a sufficient amount of drill powder was produced. This method is the most recommended.

Table 2 describes which method was used for which sample and at what time the extraction took place. After sampling, the drill powder was dried at 105 °C for about 24 h.

### 2.3. Chemical Analysis Regarding Chloride Content

The drill powder samples obtained by the methods described in Section 2.2 were then analyzed with regard to their total chloride content. For the determination of the practical total chloride content, the drill powder samples were digested using HNO_3_ cold digestion solution. The sufficiently accurate comparability of hot and cold acid digestions has already been confirmed in an inter-laboratory test conducted by the Swiss Federal Roads Office [18]. Usually, the chloride recovery rates are never 100% for all digestion methods as the complete chloride is never detected. Therefore, in the following, the chloride content decomposed by acid is considered as the total chloride content. The following procedures were used for the chloride determinations:

Procedure 1 (photometry): For the photometric determination according to DAfStb booklet 401 [19], a photometer DR 3900 from Hach Lange was used. The chloride determination was carried out according to the LCK 311 working instructions from Hach Lange. About 2.0 g of drill powder was digested with 25 mL of 18% HNO_3_ for about 10 min. The suspension was then filtered with 1.2 µm filter syringes. After filtration, 1.0 or 0.1 mL of the eluate was added to the test cuvettes, depending on the measuring range. After 3 min, the evaluation was carried out with the photometer. Depending on the chloride concentration, dilution with deionized water was used to maintain the measuring ranges of the photometer. By knowing the weight of the drill powder, the cement contents used, and a possible degree of dilution, it was possible to determine the layer-integral chloride concentration for each depth level in relation to the cement weight. Standard tests were carried out prior to the analyses, which were within the tolerance ranges specified by the manufacturer.

Procedure 2 (potentiometric titration): In this method, an eco titrator from Metrohm was used. The titration was carried out with 0.1 mol/L AgNO_3_ solution. About 2.0 g of drill powder was weighed and digested with about 5 mL of 26% HNO_3_ and about 10 mL of deionized water for about 3 min with constant stirring. Filtering of the suspension was not necessary. Titration was performed by the instrument, which independently detected the inflection point of the voltage drop. By means of a programmed formula, including the known solution consumed up to the inflection point, as well as the respective molar masses and the initial weight of the drill powder, the chloride contents related to the mass of the drill powder were thus output. By knowing the cement contents used, the chloride contents related to the mass of cement could be calculated.

The analysis methods determined approximately the same results for random analyses of the same drill powder samples. Table 2 shows which method was used for which sample.

## 3. Results and Discussion

### 3.1. Migration Coefficients and Penetration Depths Obtained in RCM Test

The migration coefficients and penetration depths obtained in the described RCM tests are shown in Table 3. The values determined are within the usual ranges for the respective binders. The slightly increased migration coefficients of series no. CIII-1 match the slightly reduced compressive strength (compared to the other concrete CEM III/A series no. CIII-2) which are most likely a result of a consistency that was slightly more fluid. 

The chloride migration coefficient D_RCM_ is strongly binder-dependent. Blast furnace cements such as CEM III/A show the greatest resistance to chloride ingress, whereas CEM I and CEM II/A-LL show the lowest resistance (see also [20,21]). The lower porosity [20,21] and smaller chloride-binding capacity [20] are stated as reasons. However, the exact consideration of the binder-dependent differences with regard to chloride penetration was not the subject of this paper.

**Figure 4:**
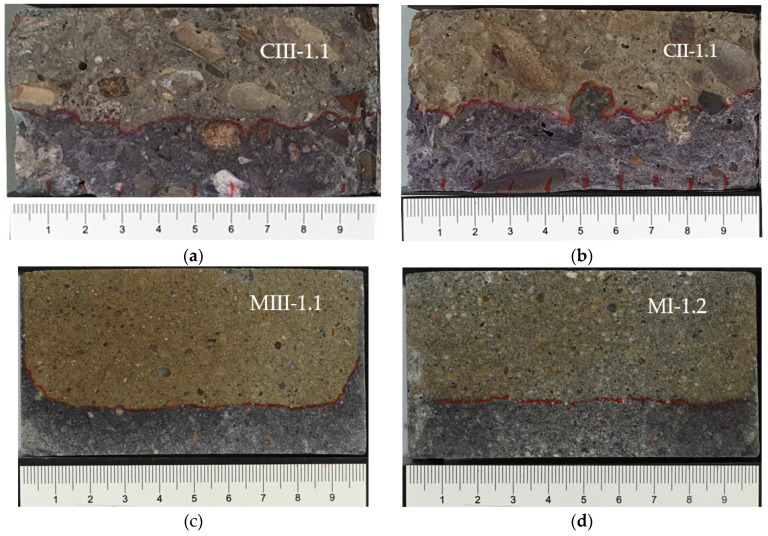
(**a**) Coloration of a CEM III/A specimen no. CIII-1.1 after AgNO_3_ application. (**b**) Coloration of a CEM II/A-LL specimen no. CII-1.1 after AgNO_3_ application. (**c**) Coloration of a CEM III/A specimen no. MIII-1.1 after AgNO_3_ application. (**d**) Coloration of a CEM I specimen no. MI-1.2 after AgNO_3_ application.

The coloration of a CEM III/A concrete specimen, a CEM II/A-LL concrete specimen, a CEM III/A mortar specimen, and a CEM I mortar specimen after AgNO_3_ application is shown in Figure 4a–d. 

**Figure 5:**
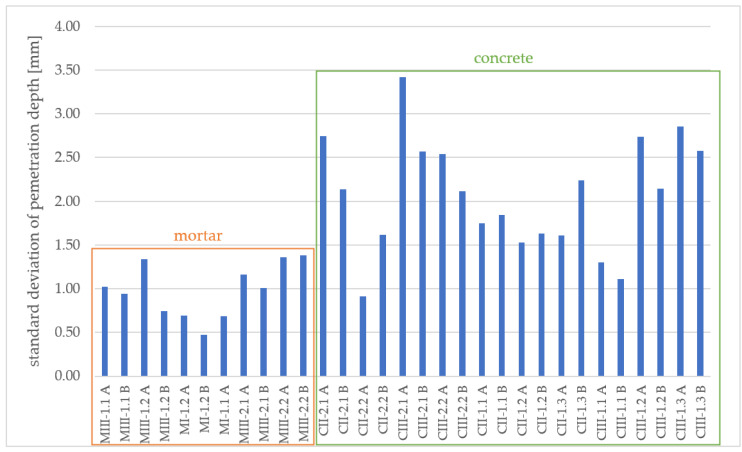
Standard deviation of penetration depth per specimen half (half A and B) measured after spraying AgNO_3_ for mortar and concrete samples.

For the concrete specimens, the penetration front is strongly irregular due to the presence of large aggregates. In contrast, the penetration front of the mortar samples is very even in general. This is also shown by the standard deviation of the penetration depth per specimen divided in half by concrete and mortar (see Figure 5). The average standard deviation of the considered mortar samples is about 1.0 mm, whereas the average standard deviation of the considered concrete samples is even, at about 2.1 mm. 

However, some concrete and mortar samples show a strong edge effect on the outer centimeter after the RCM test (Figure 4c). This means that the chlorides have penetrated deeper here than in the mid range. The reasons for this are not yet clear. Leakages between specimen and tube or increased porosities in the edge area of cylindrically cast specimens are conceivable.

### 3.2. Chloride Profiles on Concrete Samples

**Figure 6:**
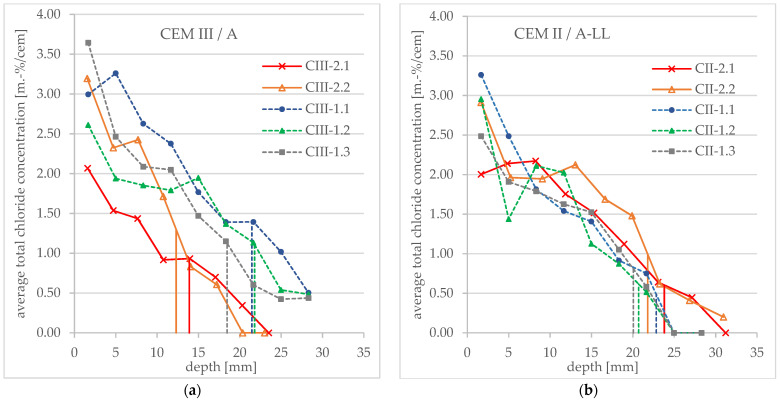
Total chloride concentration profiles obtained after an RCM test and average penetration depth (vertical lines) measured after spraying AgNO_3_ (**a**) for CEM III/A concrete specimens and (**b**) for CEM II/A-LL concrete specimens.

The obtained profiles of the total chloride concentration for the tested concrete specimens as well as the associated penetration depths are shown in Figure 6. It can be seen that the curve is much flatter and steadier compared to Tang’s model, and thus more similar to a chloride profile of a diffusion test (see Section 1 and Section 3.4). There is no plateau near the surface and a relatively shallow gradient over the depth. In addition, the curves run very unevenly. This applies to both binder types examined. 

As shown in Figure 4 and described above, the penetration front of concrete samples is often strongly irregular. When extracting concrete powder over a larger area, areas with more and less chloride content are therefore mixed and the obtained chloride profile flattens out. Due to the presence of larger aggregates, this effect can be even further intensified and lead to irregularities in the course of the profile (also known for chloride profiles taken on building site).

For this reason, chloride profiles on mortar samples with a much more regular penetration front are considered in the following. 

### 3.3. Chloride Profiles on Mortar Samples

#### 3.3.1. Overview of Obtained Chloride Profiles

**Figure 7:**
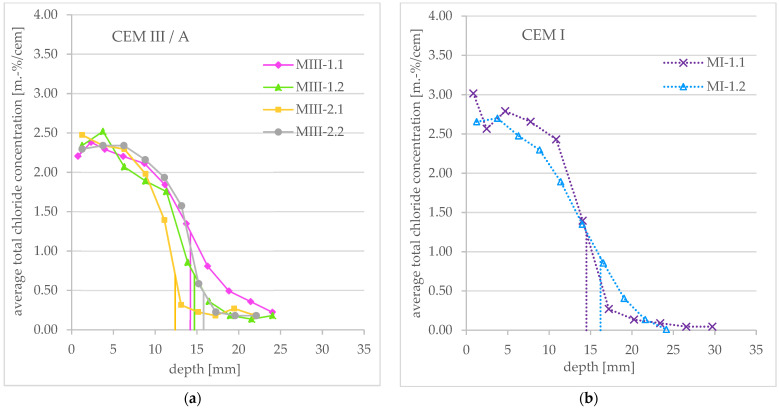
Total chloride concentration profiles obtained after an RCM test and average penetration depth (vertical lines) measured after spraying AgNO_3_ (**a**) for CEM III/A mortar specimens and (**b**) for CEM I mortar specimens.

The obtained profiles of the total chloride concentration for the tested mortar specimens as well as the associated penetration depths are shown as an overview in Figure 7. 

Compared to the profiles of the concrete samples, these profiles are much steeper and more even. A comparison with Tang’s theoretical profile is given in Section 3.4. 

In the following chapters, possible causes for the different courses of the profile within one cement type are discussed. 

#### 3.3.2. Influence of Edge Effects

**Figure 8:**
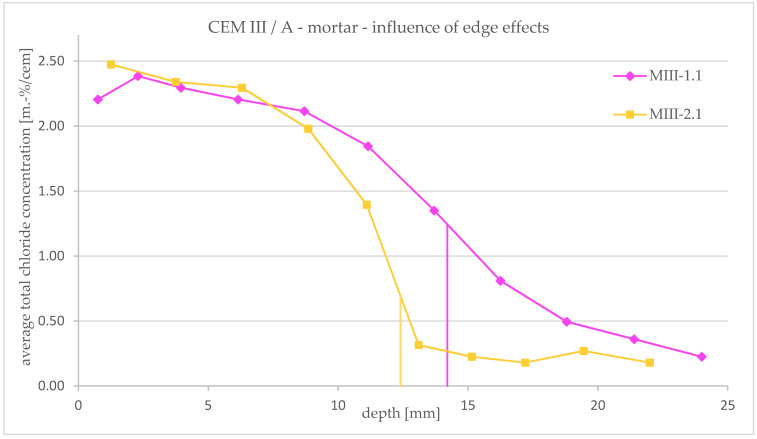
Influence of edge effects on total chloride concentration profiles obtained after an RCM test for CEM III/A mortar specimens (specimen no. MIII-1.1 analyzed concrete powder with edge effect; MIII-2.1 analyzed concrete powder without edge effect).

In Figure 8, the total chloride concentration profiles of MIII-1.1 and MIII-2.1 are shown in detail to consider the influence of the edge effect on the course of the profile. As described in Section 3.1, for some samples, there occurs a strong edge effect. If this area is included in the powder extraction amount, a mixture of different chloride concentrations takes place. This leads to a flattening of the profile as shown in Figure 8. Specimen MIII-1.1 shows this edge effect (see Figure 4c), and a large part of this edge area was included in the concrete powder extraction process (see method (c) in Section 2.2, Figure 3c). 

Specimen MIII-2.1 shows no edge effect on the one hand and on the other hand, the mortar powder was only taken from the middle area (see method (d) in Section 2.2, Figure 3d). It can therefore be assumed that the chloride content is uniform over the area under consideration and only varies in depth. Compared to the test specimen with edge effect, the profile is therefore steeper. 

The timing of the powder extraction for both samples was directly after the end of the RCM test. For the CEM I specimens, no consideration of the influence of edge effects is possible, because no mortar powder was taken from areas with edge effects for these samples. 

#### 3.3.3. Influence of Timing of Powder Extraction

**Figure 9:**
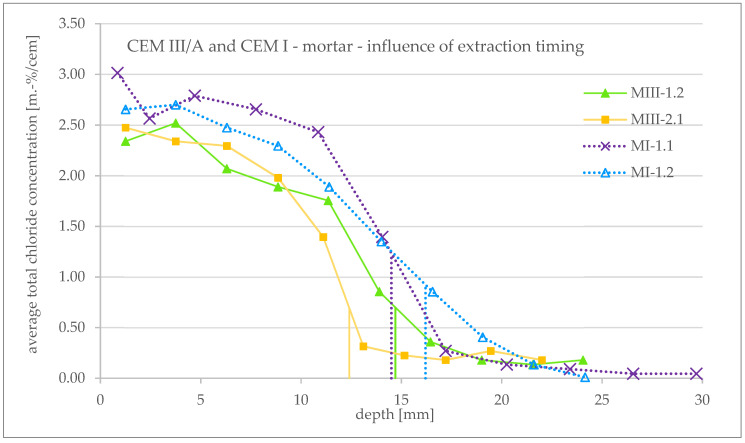
Influence of timing of concrete powder extraction on total chloride concentration profiles obtained after an RCM test for CEM III/A and CEM I mortar specimens. For specimen MIII-2.1 and MI-1.1, powder extraction was performed directly after the end of the RCM test; for MIII-1.2, extraction was performed about 3.5 weeks after the end of the test; and for MI-1.2, extraction was performed about 4.5 weeks after the end of the test.

Figure 9 shows the total chloride concentration profiles of MI-1.1 and MI-1.2 (CEM I) as well as MIII-1.2 and MIII-2.1 (CEM III/A) in detail to consider the influence of the timing of powder extraction after the end of the RCM test on the course of the profile.

For CEM III/A, the profiles of MIII-2.1 and MIII-1.2 can be compared as follows: the mortar powder of MIII-2.1 was taken directly, while the powder of MIII-1.2 about 3.5 weeks after the end of the RCM test. The latter specimen was exposed to the air at laboratory climate (≈20 °C/≈60 RH) until extraction. It can be seen that the course of the profile of specimen MIII-1.2 (late extraction) is flatter than that of MIII-2.1 (direct extraction). According to this, a further redistribution of the chlorides inside the specimen takes place after the RCM test.

The same behavior can in principle also be observed for the CEM I specimen: the profile of MI-1.2, whose powder was taken about 4.5 weeks after the end of the RCM test, runs flatter than that of MI-1.1 with a direct extraction. 

It is not yet clear whether, and if so to what extent, the binder type has an influence of the redistribution of chlorides after the RCM test. Therefore, further investigations are necessary.

### 3.4. Comparison of Measured and Theoretical Chloride Profiles

#### 3.4.1. Comparison for Concrete Samples

**Figure 10:**
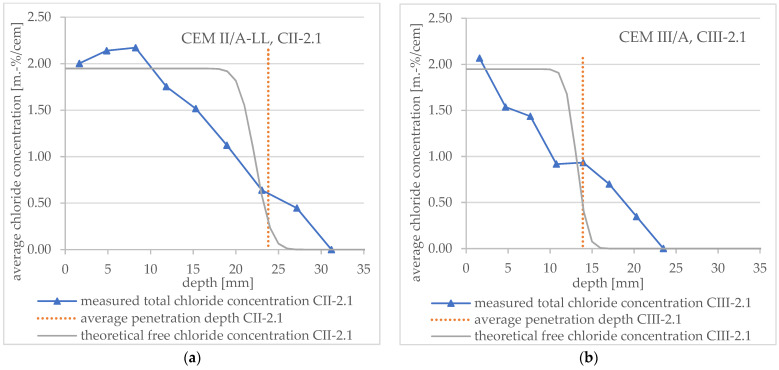
Total chloride concentration profiles measured after an RCM test including average penetration depth (vertical lines) measured after spraying AgNO_3_ and theoretical profile (**a**) for CEM II/A-LL concrete specimen no. CII-2.1 and (**b**) for CEM III/A concrete specimen no. CIII-2.1.

The following sections address the main issues regarding the comparison between the actual chloride profiles and the theoretical assumption. Figure 10 shows the measured total chloride concentration profiles of specimen CII-2.1 (CEM II/A-LL) and CIII-2.1 (CEM III/A) in detail and the theoretical free chloride concentration profiles obtained by applying Equation (3). In addition, the average penetration depth measured after spraying AgNO_3_ is presented. 

The comparison shows that the measured profiles for both binders differ significantly from Tang’s theoretical profile. In the literature, similar results can be found [7,8,9,22] (see also Section 1). A consideration and discussion of the chloride concentration at color change boundary can be found in Section 3.4.3. 

#### 3.4.2. Comparison of Mortar Samples

**Figure 11:**
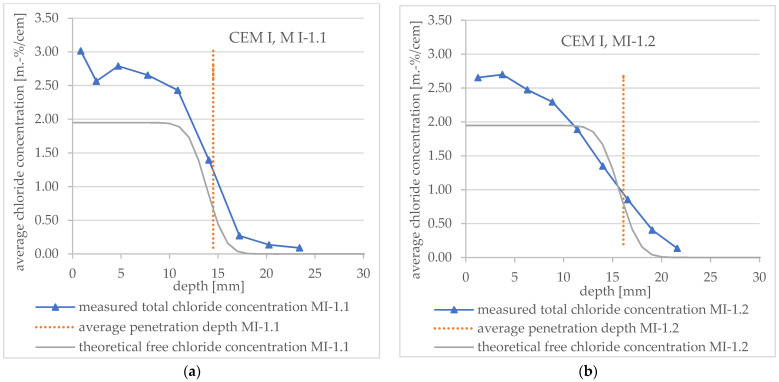
Total chloride concentration profiles measured after an RCM test including average penetration depth (vertical lines) measured after spraying AgNO_3_ and theoretical profile (**a**) for CEM I mortar specimen no. MI-1.1 and (**b**) for CEM I mortar specimen no. MI-1.2.

Figure 11 shows the measured total chloride concentration profiles of specimen MI-1.1 and MI-1.2 (both CEM I) in detail and the theoretical free chloride concentration profiles obtained by applying Equation (3). In addition, the average penetration depth measured after spraying AgNO_3_ is presented. 

**Figure 12:**
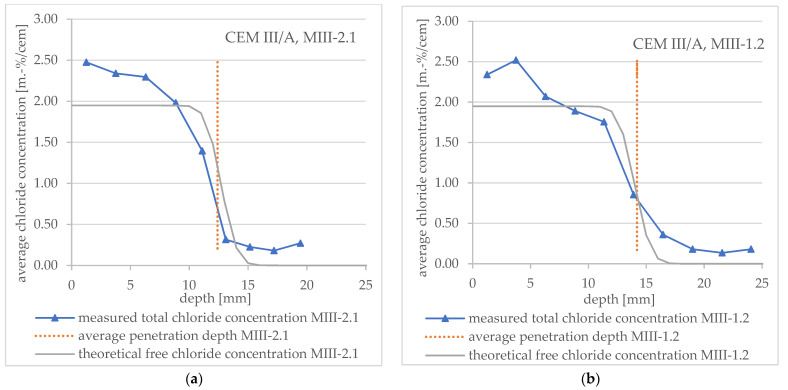
Total chloride concentration profiles measured after an RCM test including average penetration depth (vertical lines) measured after spraying AgNO_3_ and theoretical profile (**a**) for CEM III/A mortar specimen no. MIII-2.1 and (**b**) for CEM III/A mortar specimen no. MIII-1.2.

It can be seen that the slope of the measured curves and theoretical curves in diagrams 11 and 12 agree very well when the drill powder is removed directly after the end of the RCM test (Figure 11a and Figure 12a, respectively). When the drill powder is removed later (about 4.5 weeks → specimen no. MI-1.2 and 3.5 weeks → specimen no. MIII-1.2), the redistribution described above is clearly visible. The result is a deviation from the theoretical profile.

These observations apply to both cements (CEM I and CEM III/A).

#### 3.4.3. Chloride Content at Color Change Boundary

Tang [2] referred in his considerations the measurements of Otsuki et al. [23]. The latter investigated OPC specimens with different chloride concentrations and determined a free chloride concentration of 0.15 m.-% by cement at color change by pore solution expression. Tang [2] assumed a pore volume of about 0.3 mL/g cement, which leads to a chloride concentration of 0.14 mol/L. With the additional assumption, the bound chlorides escaped by the expression process, the author set a chloride concentration of c_d_ = 0.07 mol/L at color change for the calculation of the migration coefficient [2,24]. 

Figure 10, Figure 11 and Figure 12 show that the free chloride content at the color change boundary in the theoretical profiles according to Tang deviates considerably from 0.07 mol/L, respectively, about 0.07 m.-% by cement. In addition, the chloride concentration varies depending on the specimen under consideration (about 0.3 m.-% to 1.3 m.-%).

This value of 0.07 mol/L has already been tested frequently in the literature, but there is no consensus on the concentration at color change. Thus, a range of free chloride concentration from 0.01 to 0.4 m.-% by cement or 0.071 to 0.714 mol/L by pore solution, can be found [12,22,23,25,26,27]. 

The total chloride content at color change boundary, which results from the measured chloride profiles for mortar samples without edge effects, varies between about 0.5 to 1.0 m.-% by cement for both types CEM I and CEM III/A, with the exception of specimen no. MI-1.1. The reason for this exception could be that there is only one support point in the downward part of the profile, and the chloride content was therefore mixed over a greater depth. In the literature, a range of total chloride content between about 0.019 and 2.5 m.-% by cement can be found [12,22,23,25,27,28,29]. 

The data show that there is a wide scope of variability and range. Reasons given for this include sampling method, method for measuring, pH value and concentration, as well as volume of used AgNO_3_ solution [30,31,32]. There is also evidence that the chloride concentration at color change boundary varies depending on type of cement [6,33]. This is also indicated by our measured total chloride contents at the color change boundary. If this occurs, it would also have an influence on the obtained penetration depth depending on the type of binder. Further research is needed to clarify the mentioned aspects.

## 4. Conclusions

Chloride concentration profiles were investigated for mortar and concrete samples after an RCM test. Different extraction methods and times of the drilling powder were considered. In addition, the chloride penetration front was made visible by using AgNO_3_. The following conclusions can be drawn:

- The chloride profiles obtained for concrete samples are relatively flat due to the uneven penetration depth through the aggregates. The irregularity in the penetration front leads to a mixing of different chloride contents, and thus to a flattening of the profile, especially in the transition area. According to the current state of knowledge, and provided that a large area of drill powder is extracted, a steep gradient is not achievable with the means of concrete powder extraction.
- The considerations regarding chloride profiles for mortar samples have shown that the extraction method of the mortar powder, the involved area, and the timing of drill powder extraction, respectively, have a great influence on the course of the profile.
- If there are no edge effects in the area of the analyzed drill powder and the powder extraction takes place directly after the end of the RCM test, the total chloride profiles obtained on mortar samples can confirm Tang’s assumption of a steep gradient. Thus, the method of Tang to derive the chloride migration coefficient from an RCM test regarding the gradient of the chloride profile could be confirmed. Further studies on the influence of extraction timing are needed, especially to evaluate whether there are binder-dependent differences.
- With regard to the free chloride concentration at the color change boundary of the indicator, significantly higher values than the model assumption of 0.07 mol/L [2] were determined. In principle, large variations were found for both the free and the total chloride concentrations in the area of the penetration depth. Based on the investigations conducted, total chloride contents at color change boundary between 0.5 and 1.0 m.-% by cement could be found for CEM I and CEM III/A binders. Further investigations are required to be able to make a definite statement.

As a recommendation based on this research, it can be stated that precise information is required for the obtaining of chloride profiles, especially regarding the extraction of the drill powder. Local and time-dependent influences during sampling can lead to significant shifts in the profile and require further investigations. 

Further investigations are needed regarding the consideration of binder-dependent chloride binding during RCM test, and the influence of other ions in the binder systems, such as OH^−^, Na^+^, K^+^, Ca^2+^, etc. However, this was not the subject of our work.

## Figures and Tables

**Table 1:** Concrete mix design, compressive strength at the age of 28 days as well as voltage and duration of RCM test.

	Specimen	Binder	w/c	0/2	2/8	8/16	Water	Cement	Comp. Strength	Voltage	Duration
				[kg/m^3^]	[kg/m^3^]	[kg/m^3^]	[kg/m^3^]	[kg/m^3^]	[N/mm²]	[V]	[h]
concrete	CIII-1.1	CEM III/A	0.5	439.83	774.11	545.39	185.0	370.0	49.0	30	48
	CIII-1.2	CEM III/A								30	48
	CIII-1.3	CEM III/A								30	48
	CII-1.1	CEM II/A-LL	0.5	439.83	774.11	545.39	185.0	370.0	48.0	20	24
	CII-1.2	CEM II/A-LL								20	24
	CII-1.3	CEM II/A-LL								20	24
	CII-2.1	CEM II/A-LL	0.5	563	652	572	181	362	50.1	25	24
	CII-2.2	CEM II/A-LL								25	24
	CIII-2.1	CEM III/A	0.5	563	652	572	181	362	56.9	30	48
	CIII-2.2	CEM III/A								30	48
mortar	MI-1.1	CEM I	0.5	1718.9	0	0	286.5	573.0	53.3	15	24
	MI-1.2	CEM I								15	24
	MIII-1.1	CEM III/A	0.5	1718.9	0	0	286.5	573.0	46.6	30	48
	MIII-1.2	CEM III/A								30	48
	MIII-2.1	CEM III/A								30	48
	MIII-2.2	CEM III/A								30	67

**Table 2:** Extraction method and timing as well as chemical analysis method.

Specimen	Extraction Method	Extraction Timing	Chemical Analysis Method
CIII-1.1	a	ca. 3.5 weeks after RCM	Procedure 1 (see Section 2.3)
CIII-1.2			
CIII-1.3			
CII-1.1	a	ca. 3 weeks after RCM	
CII-1.2			
CII-1.3			
CII-2.1	b	directly after RCM	
CII-2.2			
CIII-2.1			
CIII-2.2			
MI-1.1	c	directly after RCM	Procedure 2 (see Section 2.3)
MI-1.2	d	ca. 4.5 weeks after RCM	
MIII-1.1	c	directly after RCM	
MIII-1.2	d	ca. 3.5 weeks after RCM	
MIII-2.1	d	directly after RCM	
MIII-2.2	d	directly after RCM	

**Table 3:** Migration coefficients (D_RCM_) and penetration depths (x_d_) obtained in RCM test.

Specimen	Binder	Migration Coefficient DRCM	Penetration Depth x_d_
		[×10^−12^ m²/s]	[mm]
CIII-1.1	CEM III/A	5.1	21.5
CIII-1.2		5.2	21.8
CIII-1.3		4.3	18.5
CII-1.1	CEM II/A-LL	16.5	22.8
CII-1.2		14.8	20.7
CII-1.3		14.6	20.0
CII-2.1	CEM II/A-LL	13.0	23.8
CII-2.2		12.4	21.8
CIII-2.1	CEM III/A	3.2	13.9
CIII-2.2		2.8	12.3
MI-1.1	CEM I	13.5	14.5
MI-1.2		15.3	16.2
MIII-1.1	CEM III/A	3.5	14.2
MIII-1.2		3.4	14.7
MIII-2.1		3.1	12.4
MIII-2.2		2.8	15.8

## Data Availability

Not applicable.
